# Second‐ or third‐generation tyrosine kinase inhibitors in first‐line treatment of chronic myeloid leukemia in general population: Is there a real benefit?

**DOI:** 10.1002/cam4.4186

**Published:** 2021-09-22

**Authors:** Jim Canet, Pascale Cony‐Makhoul, Sébastien Orazio, Edouard Cornet, Xavier Troussard, Marc Maynadié, Gabriel Étienne, Alain Monnereau

**Affiliations:** ^1^ Bordeaux Population Health Research Center Inserm UMR1219‐EPICENE University of Bordeaux Bordeaux France; ^2^ Registre des Hémopathies Malignes de la Gironde Institut Bergonié, Centre de lutte Contre le Cancer Bordeaux France; ^3^ IQVIA Bordeaux France; ^4^ FiLMC Group Centre Léon Bérard 28 Prom. Léa et Napoléon Bullukian Lyon Cedex 8 France; ^5^ Registre régional des Hémopathies Malignes de Basse‐Normandie (RRHMBN) CHU Caen Caen France; ^6^ Registre des Hémopathies Malignes de Côte‐d’Or CHU Dijon‐Bourgogne – Université de Bourgogne INSERM U1231 Dijon France; ^7^ Service d'hématologie Institut Bergonié, Centre de lutte Contre le Cancer Bordeaux France

**Keywords:** chronic myeloid leukemia, observational study, population‐based cancer registries, real life, tyrosine kinase inhibitor

## Abstract

**Introduction:**

Since 2009, multiple randomized trials have shown faster and deeper responses in CML patients treated with new‐generation TKI (NG‐TKI) compared to those treated with imatinib (IM). Are the same results observed in the general population?

**Materials and Methods:**

Patients were identified from the three French hematological malignancies population‐based registries. All CML patients (ICD‐O‐3: 9875/3) diagnosed between 2006 and 2016 and resided in registries areas were included. The TKI generation effect on achievement of MMR in first‐line therapy was assessed through a multivariate competitive risk analysis. An alluvial plot described the pathways leading to death.

**Results:**

In total, 507 CML patients received TKI in first‐line treatment, 22% were enrolled in a clinical trial. After adjustment, NG‐TKI patients were significantly more likely to achieve MMR during first‐line therapy than IM patients (HR: 1.88 CI_95%_ [1.35–2.61]). At the end of follow‐up, 212 patients were still in first‐line therapy (46 of them died), 203 switched to second‐line (43 subsequently died), 26 were on TFR from first‐line (4 subsequently died), and 20 stopped their treatment (16 subsequently died).

**Discussion:**

In this comprehensive real‐life setting, the results were consistent with clinical trials. The results are not sufficient to conclude that a NG‐TKI treatment is superior with regard to these patients, despite indications regarding differences between the TKI generation effect on survival and tolerance.

## Graphical abstract

Since 2009, multiple randomized trials have shown faster and deeper responses in CML patients treated with new‐generation TKI (NG‐TKI) compared to those treated with imatinib (IM). Are the same results observed in the general population? In this real‐life setting, the results were consistent with clinical trials: after adjustment, NG‐TKI patients were significantly more likely to achieve MMR during first‐line therapy than IM patients (HR: 1.88 CI_95%_ [1.35–2.61])

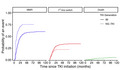

## 1 INTRODUCTION

The introduction of the first tyrosine kinase inhibitor (TKI) imatinib (IM) in 2001, followed by four other TKI up to 2013, dramatically changed the management of chronic myeloid leukemia (CML). Their impact on survival was also spectacular. In France, data from population‐based cancer registries showed a clear increase in 5‐year net survival that progressed from 49% in 1989–1993 to 83% in 2005–2010.^1^

Multiple clinical trials^2^, ^3^, ^4^, ^5^, ^6^ and meta‐analyses^7^, ^8^ have investigated the first‐line TKI treatment options and have demonstrated faster and deeper molecular response (MR) among patients treated with new‐generation TKI (NG‐TKI, i.e., dasatinib, nilotinib, bosutinib, or ponatinib) than those treated with imatinib (IM).

No effect on overall survival has been demonstrated due to the low number of events in randomized trials during the follow‐up. However, a recent meta‐analysis^7^ showed a better short‐term overall survival at 12 months after diagnosis, with a risk ratio of 0.57 CI_95%_ [0.34–0.95].

Traditional thinking in oncology concerning the results of clinical trials tends to expect a causal association between a better tumor response (described here by the molecular level of response) and longer survival,^9^ which is not observed in these trial results.

Thus, we could raise the question of the external validity of these results given the multiple concerns regarding patient selection in clinical trials and the need for long‐term follow‐up once the clinical trial has ended. Indeed, the representativeness of populations enrolled in clinical trials compared to patients treated in the general population is open to question: the literature suggests that patients enrolled in clinical trials have fewer co‐morbidities and less disease history (cardiac, pulmonary, or cancer), are younger and more rarely female.^10^, ^11^ Moreover, lower IM efficacy has been noted with patients in the general population compared to those enrolled in clinical trials.^12^

In this context, describing how trials results may be generalizable to the CML population will more accurately inform and guide physicians in the “real world.” To achieve such an objective, it is necessary to use complete non‐selected and comprehensive population‐based data.

We, therefore, aimed to estimate the effect of TKI generations on the achievement of MMR in first‐line and to describe pathways leading to death in this particular setting.

## 2 MATERIALS AND METHODS

### 2.1 Population

Cases were identified from the REPIH network composed of the three French hematological malignancies population‐based registries: the Gironde registry created in 2002, the Basse‐Normandie registry created in 1997, and the Côte‐d'Or registry created in 1982. The network provides continuous and exhaustive records on all cases of hematological malignancies in their respective geographical regions of Gironde, Calvados, Manche, Orne, and Côte‐d’Or. They covered a source population of 3,580,562 inhabitants in 2016 (5% of the French population). The National Committee of Registries certifies their quality and completeness every 4 years. In this study, all incident cases aged 18 or more at diagnosis were included, with a CML BCR‐ABL^+^ in chronic or accelerated phase (ICD‐O‐3: 9875/3) diagnosed between 1 January 2006 and 31 December 2016. Atypical CML (ICD‐O‐3: 9876/3) cases were excluded.

### 2.2 Data collection

Trained clinical research associates collected data from electronic medical reports in a standardized case report form. The phase at diagnosis was defined according to ELN 2013 criteria.^13^ The EUTOS long‐term survival score (ELTS) and Sokal score were then calculated. First‐line was defined as the first treatment for CML given after diagnosis. A major molecular response (MMR) was defined according to ELN 2013 criteria when original reports of bcr‐abl/abl value were found in medical files, or physician's interpretation of the bcr‐abl result as recorded in the patient's file if not. As molecular monitoring was performed in limited number of university labs and because of the STIC program started in France in 2005, we have considered that most of the bcr‐abl results were reports on the international scale.^14^ First‐line treatment began on the date of the first TKI prescription and ended on the date of switching to another TKI, death, last follow‐up, or beginning of a treatment‐free remission period if achieved, whichever event came first. Times to MMR and other events were calculated in months from TKI initiation. Patients not receiving first‐line TKI treatment or treated with hydroxyurea for more than 3 months were excluded. Short pre‐treatment (<3 months) with interferon alpha or hydroxyurea was allowed. Treatment dosage was not collected in the study and data were most likely not available at a satisfying level in the study population. For all patients, vital status was updated on 30 June 2018. First‐line TKI treatment was split in two groups: the “imatinib group” (IM) for patients treated with imatinib, associated or not with other treatments (i.e., interferon), and the “new‐generation group” (NG‐TKI) for patients treated with dasatinib, ponatinib, bosutinib, or nilotinib. Age was divided into three categories (<30 years old, 30–65 years old, and >65 years old), with the Eastern Cooperative Oncology Group (ECOG) score split in two (0–1 and ≥2). The care facility where the patient was first treated was dichotomized into teaching hospitals (TH) and comprehensive cancer centers (CCC) *versus* general hospitals (GH) and private clinics (PC). Adult comorbidity evaluation 27 (ACE‐27)^15^ was reported for all but one patient and split into three categories: “None,” “Mild,” and “Moderate to Severe.” The reasons for treatment switch to another line were reported and classified into three categories: “Non‐optimal response” (ELN 2013 grouping of “sub‐optimal response” and “treatment failure”), “Toxicity,” and “Other” when information was not missing and did not match the other reasons. Patients who switched for “Non‐optimal response” and “Toxicity” or “Other” were classified as “Non‐optimal response” for the analysis. We also reported if patients were enrolled in interventional clinical trials that did not investigate treatment‐free remission (TFR) strategy.

### 2.3 Analysis

**FIGURE 1:**
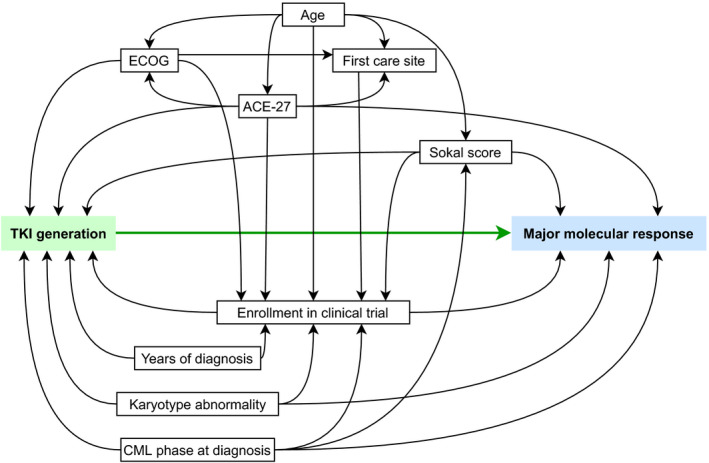
Directed acyclic graph of potential confounders for TKI generation effect on MMR in first‐line. The arrow represents the effect direction. In green exposure, in blue: outcome and in boxes: adjusted potential confounders

The primary outcome was the achievement of MMR during first‐line treatment. Secondary outcomes were reasons for treatment switch to second‐line and the death of patients. Competitive risk analysis was performed as the primary outcome was in competition with two other events: switch to another line or death. The practical approach involved in estimating the effect of TKI generations on MMR in first‐line therapy, switch to a second‐line therapy, and death in first‐line therapy with a proportional risk model where the three Cox models were adjusted for potentially confounding factors, considering competitive events and right‐censorship equivalently. Potential confounding factors were selected from clinical criteria and were included in the final model by means of a directed acyclic graph (DAG)^16^ (Figure 1). With the DAG, the following variables for adjustment in the multivariate analysis were selected: age at diagnosis (in categories), enrollment in an interventional clinical trial, care facilities where the patient was first treated, ECOG, ACE‐27, Sokal score (preferred over ELTS score as its introduction in 2016 did not match our study date), CML phase at diagnosis, presence of additional cytogenetic abnormalities at diagnosis, and the year of diagnosis. An analysis was performed on an imputed dataset. Imputation was performed by MICE (999 imputations with 10 datasets).^17^ For each incomplete variable, an imputation model was specified and different imputations per variable were created iteratively. This helped to reduce uncertainty surrounding the TKI generation effect on first‐line therapy. Risk proportionality assumptions were verified by means of Schoenfeld residuals. Patients’ characteristics were described according to their first‐line TKI generation treatment and their enrollment in a clinical trial. To facilitate interpretation of the covariable effect on the outcome, the cumulative incidence of MMR in first‐line therapy and on competitive events (first‐line switch or death) for follow‐up was plotted using the Aalen–Johansen estimator.^18^

Clinical pathways that lead to death and population characteristics according to their vital status at endpoint were described. An alluvial diagram was used to reveal the full range of disparities of these pathways.^19^ The diagram was designed to highlight patient distribution according to their clinical pathways based on chosen characteristics: that is, where patients were first treated, if they were enrolled in an interventional clinical trial, first‐line TKI, achievement of MMR during the first‐line treatment, the reason for switching to a second‐line if applicable, and vital status at endpoint. Each characteristic occupied a column in the diagram, with the columns divided into blocks corresponding to each characteristic's category. Each column is horizontally linked by flows and each flow represents a unique and specific clinical pathway. The height of the block and the size of the flow reflect the count of patients: the larger they are, the more numerous the patients. A darker color indicates the vital status at endpoint (dark: patients dead at endpoint date, light: patients alive at endpoint date). Different colors were used according to the first‐line TKI to highlight clinical pathways, with blue for IM and green for NG‐TKI. By following a flow from one column to an adjacent one, it is thus possible to study each clinical pathway.

Analyses were performed on R 3.6.3 with RStudio 1.2.5033, the following R packages were used: mice, survival, flextable, survminer, ggalluvial, and tidyverse.

## 3 RESULTS

### 3.1 Population

From 2006 to 2016, 521 CML cases in chronic phase or accelerated phase were identified. In total, 507 were included in the analysis, while 14 were excluded as they were not treated with a TKI in the first‐line (nine received hydroxyurea and five were not treated due to very old age, severe co‐morbidities, or CML in transformation). Median age at diagnosis was 61.7 year old, and 56% of patients were male. As of 30 June 2018, 18 patients (4%) transformed, 11 of them died afterward, and 98 (19%) died during follow‐up. The median follow‐up from TKI initiation was 5.1 years (Q1–Q3: 3.1–8.2 years). In the first‐line, 388 (76%) patients received IM and 109 received a NG‐TKI (90 (18%) with nilotinib, 24 (5%) with dasatinib, 3 with bosutinib, and 2 with ponatinib.

Compared to IM, patients in the NG‐TKI group were younger, had better performance status, and less severe comorbidities, with more often enrolled in clinical trials (Table 1).

**TABLE 1:** Descriptive table of the total population according to TKI generation in first‐line (*n* = 507)

Characteristics	TKI generation		Total (*n* = 507)
	IM (*n* = 388)	NG‐TKI (*n* = 119)	
Median age at diagnostic (year) – (Q1–Q3)	64.2 (52.3–74.7)	52.7 (40.3–62.6)	61.7 (49–71.7)
Age at diagnosis, *n* (%)			
<30 years	13 (3.4)	7 (5.9)	20 (3.9)
30–65 years	185 (47.6)	92 (77.3)	277 (54.7)
>65 years	190 (49.0)	20 (16.8)	210 (41.4)
Median follow‐up (year) (Q1–Q3)	5.6 (3–8.9)	5.1 (3.4–6.4)	5.1 (3.1–8.2)
Median time in first‐line (year) (Q1–Q3)	3.0 (1.3–6.3)	3.0 (1.4–5.4)	3.0 (1.3–6.0)
Sex ratio male/female	1.3	1.2	1.3
First care site, *n* (%)			
Teaching hospital and comprehensive cancer centers	292 (75.3)	100 (84.0)	392 (77.3)
General hospital and private clinic	96 (24.7)	19 (16.0)	115 (22.7)
Enrolled once in a clinical trial (%): Yes	54 (14.1)	60 (50.4)	114 (22.5)
Phase at diagnosis, *n* (%)			
Chronic	371 (95.6)	114 (95.8)	485 (95.7)
Accelerate	17 (4.4)	5 (4.2)	22 (4.3)
Sokal score, *n* (%)			
Low‐risk	100 (26.7)	49 (42.2)	149 (30.3)
Intermediate‐risk	187 (49.8)	42 (36.2)	229 (46.7)
High‐risk	88 (23.5)	25 (21.6)	113 (23.0)
*Missing*	13 (—)	3 (—)	16 (—)
EUTOS long‐term survival (ELTS) score, *n* (%)			
Low‐risk	177 (47.2)	76 (65.5)	253 (51.5)
Intermediate‐risk	138 (36.8)	26 (22.4)	164 (33.4)
High‐risk	60 (16.0)	14 (12.1)	74 (15.1)
*Missing*	13 (—)	3 (—)	16 (—)
ECOG, *n* (%)			
0–1	313 (91.5)	107 (95.5)	420 (92.5)
2–3–4	29 (8.5)	5 (4.5)	34 (7.5)
*Missing*	46 (—)	7 (—)	53 (—)
Adult comorbidity evaluation (ACE‐27), *n* (%)			
None	119 (30.7)	70 (58.8)	189 (37.4)
Mild	163 (42.1)	38 (31.9)	201 (39.7)
Moderate to severe	105 (27.1)	11 (9.2)	116 (22.9)
*Missing*	1 (—)	0 (—)	1 (—)
Deceased at 30 June 2018, *n* (%)	104 (26.8)	5 (4.2)	109 (21.5)

The characteristics of the 114 patients (22%) enrolled in the clinical trial are presented in Table 2. As expected, patients enrolled in clinical trials were younger, had fewer comorbidities, and were all first treated in a teaching hospital or a comprehensive cancer center.

**TABLE 2:** Descriptive table of the population according to enrollment in a clinical trial at diagnosis (*n* = 506, one missing data for a patient)

Characteristics	Not enrolled in a clinical trial (*n* = 392)	Enrolled in a clinical trial (*n* = 114)
Median age at diagnostic (year) – (Q1–Q3)	63.2 (51.2–73.9)	57.1 (41.8–64.4)
Age at diagnosis, *n* (%)		
<30 years	14 (3.6)	6 (5.3)
30–65 years	197 (50.2)	80 (70.1)
>65 years	181 (46.2)	28 (24.6)
Median follow‐up (year) (Q1–Q3)	5.3 (3.0–8.1)	6.2 (3.8–9.7)
Median time in first‐line (year) (Q1–Q3)	2.9 (1.2–6.0)	3.8 (1.9–6.0)
Sex ratio male/female	1.2	1.5
TKI, *n* (%)		
IM	333 (84.9)	54 (47.4)
NG‐TKI	59 (15.1)	60 (52.6)
First care site, *n* (%)		
Teaching hospital and comprehensive cancer centers	278 (70.9)	114 (100.0)
General hospital and private clinic	114 (29.1)	0 (0.0)
Phase at diagnosis, *n* (%)		
Chronic	372 (94.9)	112 (98.2)
Accelerate	20 (5.1)	2 (1.8)
Sokal score, *n* (%)		
Low‐risk	111 (29.3)	38 (34.2)
Intermediate‐risk	182 (48.0)	47 (42.3)
High‐risk	86 (22.7)	26 (23.4)
*Missing*	13 (—)	3 (—)
EUTOS long‐term survival (ELTS) score, *n* (%)		
Low‐risk	193 (50.9)	60 (54.1)
Intermediate‐risk	132 (34.8)	32 (28.8)
High‐risk	54 (14.2)	19 (17.1)
*Missing*	13 (—)	3 (—)
ECOG, *n* (%)		
0–1	314 (91.8)	106 (95.5)
2–3–4	28 (8.2)	5 (4.5)
*Missing value*	50 (—)	3 (—)
Adult comorbidity evaluation (ACE‐27), *n* (%)		
None	130 (33.2)	59 (51.8)
Mild	156 (39.9)	45 (39.5)
Moderate to severe	105 (26.9)	10 (8.8)
*Missing*	1 (—)	0 (—)
Deceased at 30 June 2018, *n* (%)	100 (25.5)	8 (7.0)

### 3.2 First‐line response

First‐line MMR data were missing for 4% (21/507) of patients (19 IM and 2 NG‐TKI). With first‐line treatment, 316 patients (316/486, 65%) achieved MMR within a median time of 10.5 months (median; Q1–Q3: 6.0–18.0 months). Seventy‐nine percent (89/112) of patients enrolled in clinical trials achieved MMR *versus* 61% (227/392) of those not enrolled. Patients treated with NG‐TKI achieved MMR more often and faster than patients treated with IM, respectively, 77% (90/117) in a median time of 6.0 months (Q1–Q3: 6.0–12.0 months) and 61% (226/369) in a median time of 12.0 months (Q1–Q3: 7.8–20.0 months).

To perform competitive risk analysis, data were imputed for five variables with missing values (enrollment in a clinical trial, performance status, comorbidity severity, presence of additional cytogenetic abnormalities at diagnosis, and Sokal score). Data were not imputed for MMR status in first‐line and time to achieve MMR in first‐line (Table S1).

**FIGURE 2:**
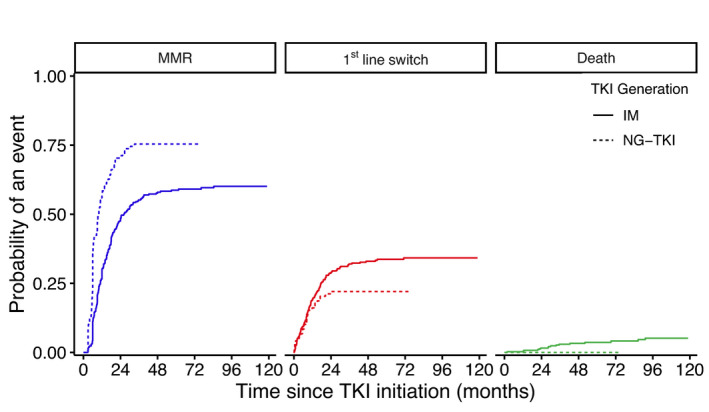
Cumulative incidence of MMR (blue) or death (green) or first‐line switch (red) according to treatment generation from 0 to 120 months after treatment initiation

Following adjustment on the potential confounding factors identified, patients in the NG‐TKI group were significantly more likely to achieve MMR during first‐line treatment than those in the IM group (HR: 1.88 CI_95%_ [1.35–2.61]) (Table 3). After visual verification, non‐linearity of hazard risk assumption over time was rejected for all variables. The results were in line with those displayed by the cumulative incidence analyses (Figure 2).

**TABLE 3:** Estimated cause‐specific hazard ratio (and 95% confidence interval) of the effect of TKI generation on first‐line MMR, first‐line switch and survival in first‐line using multivariate Cox regression model in a competitive risk context (NG‐TKI, new‐generation tyrosine kinase inhibitor; IM, imatinib)

	Cause‐specific hazard ratio		
	MMR	Line switch	Death
NG‐TKI *versus* IM	1.88 [1.35–2.61]	1.14 [0.68–1.90]	—^a^

^a^ Model did not converge.

### 3.3 Switching and discontinuation of first‐line and survival

At the end of follow‐up, 42% (212/507) of patients were still in first‐line and 9% (46/507) died while in first‐line, 40% (203/507) switched to a second‐line of treatment and 21% (43/203) subsequently died, 5% (26/507) were on TFR from first‐line and 15% (4/26) subsequently died, and 4% (20/507) stopped their first‐line treatment without starting a second‐line of whom 80% (16/20) subsequently died. The characteristics of deceased patients (*n* = 109) are described in Table 4. All but five were treated with IM in first‐line. Deaths occurred after a median follow‐up of 3.3 years (2.0–6.3). As expected, patients who died were older at diagnosis (median of 73.2 years vs. 58.3 years for those alive at the end of follow‐up) and none were under 30 years. They had higher risk scores (especially ELTS score), a bad performance status at diagnosis (19.4% with ECOG ≥2 vs. 4.4%), more comorbidities (83.4% with ACE27 mild to severe vs. 56.9%), and were less frequently treated in TH or CCC (68.8% vs. 79.6%). 61% (66/109) of the deceased patients had not switched to a second‐line, 39% (26/66) had not achieved MMR, all but one were treated with IM, and 42% (11/26) were treated in GH or PC (vs. 23% in the study population). Competitive risk analysis on TKI generation effect on survival in first‐line therapy did not converge, as events were too few (26 events and all but one in the IM group), thus result in this setting was not available (Table 3).

**TABLE 4:** Characteristics of the population according to their vital status at the end of follow‐up (*n* = 507)

Characteristics	Alive (*n* = 398)	Dead (*n* = 109)
Median age at diagnostic (year) – (Q1–Q3)	58.3 (46.5–68.7)	73.2 (64.1–80.4)
Age at diagnosis, *n* (%)		
<30 years	20 (5.0)	0 (0.0)
30–65 years	248 (62.3)	29 (26.6)
>65 years	130 (32.7)	80 (73.4)
Median follow‐up (year) (Q1–Q3)	6.0 (3.7–9)	3.4 (2.1–6.4)
Median time in first‐line (year) (Q1–Q3)	3.3 (1.6–6.3)	2.0 (0.8–4.2)
Sex ratio men/women	1.3	1.3
First care site, *n* (%)		
Teaching hospital and comprehensive cancer centers	317 (79.6)	75 (68.8)
General hospital and private clinic	81 (20.4)	34 (31.2)
Sokal score, *n* (%)		
Low‐risk	129 (33.5)	20 (18.9)
Intermediate‐risk	169 (43.9)	60 (56.6)
High‐risk	87 (22.6)	26 (24.5)
*Missing*	13 (—)	3 (—)
EUTOS long‐term survival (ELTS) score, *n* (%)		
Low‐risk	221 (57.4)	32 (30.2)
Intermediate‐risk	118 (30.6)	46 (43.4)
High‐risk	46 (11.9)	28 (26.4)
*Missing*	13 (—)	3 (—)
ECOG, *n* (%)		
0–1	345 (95.6)	75 (80.6)
2–3–4	16 (4.4)	18 (19.4)
*Missing*	37	16
Adult comorbidity evaluation (ACE‐27), *n* (%)		
None	171 (43.1)	18 (16.5)
Mild	158 (39.8)	43 (39.4)
Moderate to severe	68 (17.1)	48 (44.0)
*Missing value*	1 (—)	0 (—)
Reason for switch^a^, *n* (%)		
Non optimal response	85 (53.1)	29 (67.4)
Toxicity	64 (40.0)	14 (32.6)
Other	11 (6.9)	0 (0.0)

^a^ Only concerning the 203 patients that had switched to second‐line of treatment.

**FIGURE 3:**
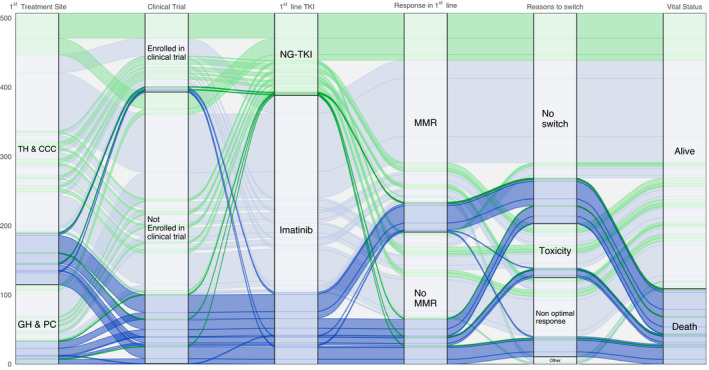
Alluvial plot representing flows of patients since diagnostic to endpoint. Green flows represent NG‐TKI‐treated patients and blue flows represent IM‐treated patients. Flows are represented according to where patients were first treated, they enrollment in a clinical trial, their TKI generation prescribe in first‐line, their MMR achievement during first‐line, the reason for second‐line switch if any, and vital status at end point. In dark blue and dark green flows leading to death and in light blue and light green flows leading alive patients at the end of follow‐up. CCC, comprehensive cancer center; GH, general hospital; MMR, major molecular response (*n* = 507); NG‐TKI, new‐generation TKI (i.e. other than IM); PC, private clinic; TH, teaching hospital

To illustrate patients’ pathways and visualize their characteristics, an alluvial plot was drawn according to: the first care facility, whether they were enrolled in a clinical trial, first‐line TKI generation, whether they achieved MMR during first‐line treatment, reasons for treatment switch if appropriate, and final vital status (Figure 3).

The alluvial diagram revealed 59 different clinical pathways, with 1–84 patients in each. Eighteen pathways, representing 109 patients, led to death (darker color). The largest involves 25 patients. It illustrates patients that were still in first‐line treatment, first treated in TH or CCC with IM, and achieved MMR before death.

More generally, the diagram illustrates the different characteristics of deceased patients. It shows they were more frequently treated in GH or PC than TH or CCC, were not enrolled in a clinical trial, were treated in first‐line with IM (all but 5), and had equivalently achieved MMR or switched treatment line. Deceased patients that did not switch to another line can be dichotomized into groups of patients that achieved MMR (40/66) or not (26/66). Their median time from treatment initiation to death was, respectively, 4.4 years (Q1–Q3: 3.1–6.5) and 2.3 years (Q1–Q3: 1.4–3.5). Among the deceased patients, 60 out of 75 that did not achieve MMR in the first‐line and 11 of the 34 that did not switch to a subsequent line were treated in GH or PC. The median time from treatment initiation to death was, respectively, 2.0 years (Q1–Q3: 1.5–2.8) and 2.6 years (Q1–Q3: 1.4–6.5). As expected, deceased patients switched proportionately more frequently from first‐line due to a non‐optimal response.

Regarding the group of patients that switched to second‐line (*n* = 203), the main reason for switching was a non‐optimal response (56%, 114/203), followed by toxicity alone (78/203, 39%), while 5% of patients (11/203) switched for unspecified reasons. A switch to a second‐line occurred for 41% patients (160/388) in the IM group and 36% (43/119) in the NG‐TKI group. The main reasons for switching were toxicity in the NG‐TKI group (58% (25/43) vs. 33% (53/160) in the IM group), and a non‐optimal response in the IM group (61% (98/160) vs. 37% (16/43) in the NG‐TKI group). Following adjustment on the potential confounding factors identified, no significant effect of TKI generation on treatment switch was found in first‐line therapy while in competition with MMR or death (HR: 1.14 CI_95%_ [0.68–1.90]) (Table 3). After visual verification, non‐linearity of hazard risk assumption over time was rejected for all variables. The results were in line with those displayed by the cumulative incidence analyses (Figure 2).

Among the group of patients (*n* = 20) that stopped their first‐line treatment without switching, 80% (16/20) were treated with IM, 63% (10/16) stopped the first‐line for an unknown reason, 9 subsequently died, and 37% (6/16) experienced a blastic phase and subsequently died. Regarding the four NG‐TKI patients, one died, while three experienced a blastic phase and were still alive at the end of follow‐up.

Among the 26 patients in TFR from the first‐line, 77% (20/26) were treated with IM, 4 of whom died afterward while in MMR or in deeper response, and 23% (6/26) were treated with NG‐TKI. All but five IM patients were treated in CCC or TH with a median time of 6.0 years (Q1–Q3: 4.7–7.4).

## 4 DISCUSSION

In this population‐based study, patients treated with NG‐TKI were more likely to achieve MMR in first‐line therapy at all times than patients treated by IM (HR: 1.88 CI95% [1.35–2.61]). This result is consistent with clinical trial results^2^, ^3^, ^4^, ^5^, ^6^ and with other population‐based studies. In 2015, Di Bella et al. reported a better rate of MMR after 6 months of treatment among 78 NG‐TKI patients from 222 IM patients. In 2017, Hoffmann et al. used the EUTOS population‐based registry to note a better incidence rate of MMR at all times in NG‐TKI patients. The UK TARGET CML study in 2020 also described a better rate of MMR in the first‐line among NG‐TKI patients (63% against 50% for IM patients). Finally, in 2017, Geelen et al. described a faster MMR rate among patients treated by NG‐TKI in the first‐line.^20^, ^21^, ^22^, ^23^

One of the scientific justifications for this study was to examine the representativeness of populations enrolled in clinical trials in relation to the population reached, that is, the population actually treated with TKIs. As expected, divergences were found in the study population between the patients enrolled and not enrolled in clinical trials: enrolled patients represented 22% of the treated population, were younger, and had a better overall condition and less severe co‐morbidities than non‐enrolled patients. These differences were also found by Latagliata et al.^24^ among patients non eligible for both DASISION and ENESTnd clinical trials. This justifies the interest of exploring the difference in relative effect of the two generations of TKIs used in first‐line treatment in the general population.

Those differences described could partly explain characteristics dissimilarity observed among NG‐TKI patients since 50% of them were included in a clinical trial compared to 14% for IM patients. Another explanation could be the availability of the treatment at the time of diagnosis: no NG‐TKI in first‐line were available in France before 2011 outside clinical trials and, after, only nilotinib was reimbursed by the French social security, and to the choice of the practitioner who may have favored NG‐TKI for a population with a better overall condition. Those results diverged from the Italian SIMPLICITY cohort^25^ and the UK TARGET CML population^22^ where NG‐TKI patients had a Sokal score risk higher than IM patients. In the SIMPLICITY cohort, NG‐TKI patients were older than IM patients and NG‐TKI were only prescribed in academic center. In the UK TARGET CML study, the overall age at diagnosis was younger than in our population and with NG‐TKI patients younger than IM patients.

It was observed that 41% of patients treated with IM in first‐line switched to a second‐line. This proportion was 36% for patients treated with NG‐TKI. These results differ from Hoffmann^20^ where lower switch rates were found for IM and NG‐TKI patients (28% and 20%, respectively). This could be explained by the patients’ temporality and country of origin where other treatments may not have a market authorization. However, they are in line with the study by Castagnetti et al.^26^ that focused on IM only showing a 41% switch rate. Our results were also close to the Milojkovic et al. 2020 study^22^ with a 45% switch rate for IM and 41% for NG‐TKI.

Our study also examined patients’ individual trajectories, identifying common characteristics in deceased patients from a descriptive point of view only. Indeed, given the small size of this sub‐population, comparison of treatments on survival is not statistically feasible. The alluvial plot already presents a wide variety of clinical pathways that illustrate the complexity of how CML patients are treated, using only six variables and illustrating the need for patients to be strictly followed to ensure the most efficient response to treatment, since the majority of deceased patients did not respond to their first‐line treatment. The study also unexpectedly described some non‐MMR deceased patients who had not switched to a second‐line after 1 year of treatment. This may be explained by practitioners’ desire to avoid adverse effects of NG‐TKI on these patients, or by poorer patient follow‐up, or simply that practitioners evaluated the disease as under control even if MMR was not yet achieved, or that they had not been able to follow treatment guidelines.^27^ In addition, proportionally more patients died in GH or private clinics than in CCC and TH. One possible explanation is that age, ECOG status, and comorbidities are probably linked to the first care site of treatment: that is, only patients in better condition are referred to TH or CCC, and TH and CCC physicians probably have more experience in CML than those working in GH or private clinics.

MMR was described in a real‐life setting as a prognostic factor for patients’ outcomes which is consistent with the literature,^28^, ^29^ confirming the choice of MMR as the main outcome criterion for our study.

Moreover, only five deceased patients had first‐line treatment with a NG‐TKI, which can be explained by the lower proportion of patients treated by NG‐TKI, by shorter follow‐up, by patients’ characteristics: that is, age, ECOG, and ACE‐27, or by enrollment status in a clinical trial.

The same superiority of NG‐TKI over IM in the achievement of MMR in first‐line was not noted for survival. The results point to an improvement in survival, however, despite the lack of data to evaluate it.

Other parameters are needed to assess the overall effectiveness of TKI, such as the patient's tolerance to treatment. Indeed, treatment toxicity is the primary reason for switching in the NG‐TKI group.

However, no overall difference was found between TKI generation on treatment switch before MMR or death. Nevertheless, the second‐generation TKI appears to be more effective, but also more toxic in a first‐line context and, therefore, its first‐line indication appears to require better selection of eligible patients.

The study presented data on the external validity of clinical trials, comparing the first‐line TKI generation in CML and assessing the different treatment effects in the population reached. It was not a substitute for rigorous clinical trials and did not estimate the difference in efficacy between the TKI generations. The study has both strengths and limitations. Its major strength is its comprehensiveness and absence of population selection. Moreover, the REPIH network's expertise allowed us to identify all CML case incidents and to ensure optimal quality of the information extracted from the medical records, thereby reducing the biases inherent in the retrospective and observational nature of our study. This resulted in a reduced amount of missing data, with only five of the variables used for adjustment being partially incomplete. 25% of the patients lacked data for one variable. The MICE method was thus applied to avoid reducing the power arising from a complete case analysis. Given the observational nature of our study, the results need to be qualified. To control for confounding by indication bias, the TKI generation effect on achieving MMR in first‐line therapy was adjusted for potential confounding factors selected according to their “clinical relevance.” As the treatment strategy evolved (ELN recommendations or evidence of TKI‐specific adverse effects) and prescription practices changed during the study period, the result was also adjusted over the course of the calendar year of the treatment. Similarly, in order to correct for treatment effect, a “simple” adjustment approach was preferred. Given the limited number of adjustment factors and the large number of events that occurred, use of more complex methods such as inverse ponderation weighting of patients in the population by propensity scores^30^, ^31^ could have been adopted. However, we feel that such methods would not have been more efficient and would have made it more difficult to interpret the results. In addition, it was not possible to consider potential interval censorship in the survival analysis: patients were not followed "continuously" but on a regular basis, generally every 3 months after initiating treatment, and then less regularly after a year. Thus, in line with clinical trial practices, the date of achieving MMR was considered as the date of MMR assessment by the practitioner.

To conclude, in this comprehensive CML cohort representative of real life, with robust statistical methods, our study showed that incident CML patients with NG‐TKI as the first‐line treatment were more likely to achieve MMR than populations treated with IM first‐line. These observations are consistent with clinical trial results despite the disparities in the CML population, whether enrolled or not. However, our results cannot conclusively point to the superiority of NG‐TKI treatment regarding these patients, despite indications regarding differences between the TKI generation effect on survival or tolerance. At the population level, the study showed that there were discrepancies between patients by treatment site, that less than half of patients progressed to a second‐line, mostly for a sub‐optimal response, and that TFR was rarely implemented in first‐line during follow‐up.

## ETHICS STATEMENT

Data collection and analyses were performed within French National Commission on Informatics and Liberty (*CNIL*) authorization scope of the three registries participating in the study (*Registre des Hémopathies Malignes de Côte d’Or: CNIL n^o^ 97.013*, *Registre des hémopathies malignes de la Gironde: CNIL n^o^ 90.3445*, and *Registre Régional des Hémopathies Malignes de Basse‐Normandie: CNIL n^o^ 1276682*).

## CONFLICT OF INTEREST

Dr. Cony‐Makhoul reports grants from Force Hémato, during the conduct of the study. Other authors declare no potential conflict of interest related to this study.

## AUTHOR CONTRIBUTION

JC, AM, and PCM conceived of the presented study. JC and PCM wrote the present article helped by AM. JC developed the theory and performed the analyses, SO discussed the analytical methods. AM, SO, MM, EC, and XT supervised the data collection. PCM, AM, and GE gave guidance to JC on the findings to be presented and the discussion. All authors discussed the results and contributed to the final manuscript.

## Supporting information

Table S1

## Data Availability

The data that support the findings of this study are available upon request from the corresponding author after contracting with all the registries participating in the study. The data are not publicly available due to privacy or ethical restrictions.
