# Supplementary material for: Second‐ or third‐generation tyrosine kinase inhibitors in first‐line treatment of chronic myeloid leukemia in general population: Is there a real benefit?
Source: Cancer Med. 2021 Sep 22;10(20):6959–70. doi: 10.1002/cam4.4186 (PMC8525157; doi:10.1002/cam4.4186)
Supplement: Supplementary file 1 — Table S1 [file CAM4-10-6959-s001.docx]

# Supplementary Data

Supplementary Table 1. Table of missing value in the population (n = 507)

|  | **Missing Value (%)** | |
| --- | --- | --- |
| MMR during first line | 21 | (4.1) |
| Time to MMR in first line* | 10 | (3.1) |
| Enrollment in a clinical trial | 1 | (0.2) |
| Performance status (ECOG) | 53 | (10.5) |
| Adult Comorbidity Evaluation (ACE 27) | 1 | (0.2) |
| Sokal score | 16 | (3.2) |
| Additional Cytogenetic abnormalities | 51 | (10.1) |
| *Missing value counted for patients in MMR (n = 316) | | |
